# Implementation of a Positive Youth Development Program in a Chinese Context: The Role of Policy, Program, People, Process, and Place

**DOI:** 10.1100/tsw.2008.120

**Published:** 2008-10-10

**Authors:** Daniel T. L. Shek, Rachel C. F. Sun

**Affiliations:** ^1^ Quality of Life Centre, Hong Kong Institute of Asia-Pacific Studies, The Chinese University of Hong Kong, Hong Kong; ^2^ Kiang Wu Nursing College of Macau, Macau; ^3^ Social Welfare Practice and Research Centre, The Chinese University of Hong Kong, Hong Kong

**Keywords:** adolescence, positive youth development, education, school, project implementation, Project P.A.T.H.S.

## Abstract

In this paper, a case study is presented based on one school, conducted to examine the factors that influenced the process and quality of implementation of the Tier 1 Program of the Project P.A.T.H.S. Through interviews with the school contact person and focus group interviews with the instructors, an integration of the findings showed that several factors related to the program, people, process, policy, and place (5 “P”s) facilitated the implementation process of the Tier 1 Program in the school. Obstacles and difficulties with reference to the 5 “P”s that affected the quality of implementation were also identified. Overall, the quality of program implementation in the school was good, and the program was well received by the program implementers. Implications of the present findings for future program implementation with reference to school administrative arrangements and implementation issues are discussed.

## INTRODUCTION

"P.A.T.H.S. to Adulthood: A Jockey Club Youth Enhancement Scheme" is a holistic positive youth development program developed for junior secondary school students in Hong Kong. The word "P.A.T.H.S." denotes **P**ositive **A**dolescent **T**raining through **H**olistic **S**ocial Programmes[1,2], with two tiers of programs. While the Tier 1 Program is a universal program that utilizes a curricula-based approach for all Secondary 1 to 3 students of the participating schools, the Tier 2 Program is provided for students who have greater psychosocial needs.

For the Tier 1 Program, each participating school has the freedom to determine who should teach the program (e.g., implementation by teachers and/or social workers), the degree of incorporation into the formal curriculum (e.g., Life Education, Liberal Studies, Moral and Civic Education, Religious Studies, and Integrated Humanities), the use of different time slots (e.g., form teachers' period, morning assembly, weekly assembly, after school period, Saturday mornings, extracurricular activities, and day camps), and the combination of different teaching modes[3].

Despite the importance of implementation quality in positive youth development programs, systematic research in this area is inadequate. Fagan and Mihalic[4] criticized that “much attention has focused on identifying effective research-based programs. In contrast, there has been much less awareness of the factors needed to successfully implement such programs, even though successful implementation is not guaranteed by adopting a best practice program” (p. 236). In their review of the existing school prevention programs, Elias et al.[5] pointed out that effective program implementation consists of several elements. The first element is a good program – the program should be well designed with clear goals, should be tailor made for the adolescents’ needs, should be socially and culturally sensitive, should be user friendly, should have ready-made teaching materials, and should teach practical knowledge and generic skills. The second element is good administrative arrangements – the school fits the program into the school policy, carries it out at an appropriate time of adolescent development, allows sufficient time for implementation and follow-up, has a nonauthoritative leadership and clear assignment of roles and responsibilities for workers, and has periodic evaluation with revision. The third element concerns the implementation process, where teachers are trained before teaching, and multiple teaching strategies, updated materials, multimedia teaching aids, and incentives are used. Similar characteristics of effective program implementation have also been advocated by other researchers[6,7,8].

While factors that significantly contribute to program implementation quality have been identified in previous studies, Kam et al.[9] criticized that “previous implementation studies have usually examined single facets of implementation and have failed to look at the interrelationship among multiple facets of implementation” (p. 59). In addition, Liljia et al.[7] stressed that “to improve the development of prevention programs, it is a clear advantage to have a model of the factors and the main mechanisms that lead to courses having beneficial effects” (p. 1850).

To fill the existing research gap, factors that influence the quality of program implementation were examined in this study with respect to five aspects of program implementation, including program, people, process, policy, and place (5 “P”s). First, program content focused on practical knowledge, competencies, and generic skills, and program design that was evidence based was conducive to the instructors’ willingness to implement the program as intended, which in turn contributed to effective program implementation[10]. On the other hand, program activities that overlapped with other existing programs in the school were more likely to make the students feel bored, which negatively affected the program implementation[4]. In short, programs that have novel and attractive activities are preferable.

Second, studies showed that school principals, coordinators, and teachers (i.e., people) contributed to the effectiveness of school-based prevention programs[4,9]. The principal’s leadership was found to be a significant factor affecting program success[11]. In addition, committed coordinators who offered ongoing support to the front-line teachers, fostered communication among different parties, and provided clear guidance and information regarding the program implementation contributed to effective program implementation[4].

Furthermore, studies showed that teachers who had been trained to teach were vital because training workshops could familiarize teachers with the program philosophy and content, the necessary teaching strategies, the need for fidelity, as well as cultivating their enthusiasm and support for the program[4,12], which in turn contributed to program implementation quality[10,11]. Research studies also showed that characteristics of the teachers, including abundance of teaching experience, good preparation, possession of a graduate degree, and a greater sense of belonging to the school contributed to program effectiveness[10,13].

Concerning the program implementation process, effective delivery, such as using interactive teaching strategies[10] and facilitation skills to teach the program concepts and skills, and to generalize the students’ learning across the school day[9], significantly contributed to the effectiveness of school-based prevention programs. In addition, adherence to the designed program as well as good classroom and time management skills were highlighted to be the key process factors of quality implementation[4]. Kam et al.[9] stressed that support of the principal in the implementation process was necessary in order to promote effective delivery because it could encourage teachers to devote themselves to the program implementation. Consequently, teachers were more enthusiastic to teach and likely to create a positive classroom atmosphere that raised the students' learning interest and motivation[4].

Concerning the school policy, the schools that integrated the program into their formal curriculum and emphasized program fidelity were found to have effective program implementation[4]. The formulation of clear goals in the program implementation process also provided a clear direction for the program implementers[11]. Furthermore, good manpower deployment with division of labor was crucial to prevent teacher burnout due to overloaded teaching and administrative duties[11]. Researchers also suggested that the school arrange regular meetings with the teachers to discuss problems encountered and the respective solutions, and to build a supportive working environment as well as organize final evaluation meetings to applaud good work done, review past practices, and gain feedback[4].

Finally, a warm school environment ("place"), where the school principal, coordinators, and teachers are supportive to each other and who think along the same line in supporting the implementation of the program in the school, is conducive to effective program implementation. In addition, a nonauthoritarian working environment that allows collaborative decision making and a positive atmosphere that enables job satisfaction facilitates effective program implementation[13,14]. Furthermore, Eccles and Gootman[15] listed several physical features that promoted positive youth development, including settings characterized with physical and psychological safety, appropriate structure, supportive relationships, opportunities to belong, positive social norms, support for efficacy, opportunities for skill building, and integration of family, school, and community efforts.

Yin[16,17] discussed the strengths of case study research. In this study, a case study was conducted in order to understand the contribution of the above factors to the quality of program implementation in the Chinese culture[11]. There were two objectives of the case study: (1) to investigate the facilitating factors and obstacles encountered in the program implementation process from the program implementers' perspectives and (2) to draw implications for future program implementation. Specifically, three aspects of the program implementation (including administrative arrangements, implementation characteristics, and perceived program evaluation and success) were examined in light of the five categories of factors (i.e., program, people, process, policy, and place) that influence program outcomes.

## METHODS

### Participants

A total of 207 schools joined the Project P.A.T.H.S. (Secondary 1 Curriculum) in the Full Implementation Phase in the 2006/07 academic year. In the Tier 1 Program, 112 of these schools adopted the full program (i.e., 20-h program involving 40 teaching units) and 95 adopted the core program (i.e., 10-h program involving 20 teaching units). Among these participating schools, six schools that joined the full program and one school that joined the core program were invited to participate in the case study. Based on the convenience sampling method, one school where the Tier 1 Program was delivered in the class teachers' period and other subjects was selected for this case study.

### Procedures

In this case study, an individual interview with the school contact person and a focus group interview with six instructors of the Tier 1 Program of the Secondary 1 Curriculum were conducted separately. Prior consent from the principal was sought for the school's participation. At the beginning of the interviews, informed consent was also obtained from the respondents, and the purpose of the study and the principle of confidentiality were emphasized repeatedly. The second author and a trained colleague with a Master degree in Counseling conducted the interviews. As the purpose of this case study was to examine the school administrative arrangement and its implementation process of the Tier 1 Program (Secondary 1 Curriculum) in a retrospective perspective, the interviews were conducted in mid-June 2007, after the school had completed the Tier 1 Program. Specific self-constructed semi-structured interview guides for the individual interviews and the focus group interviews were designed to collect the data. There were several parts in each interview guide:

- Respondents' perceptions of the school administrative arrangement (including preparation, support within the school, and from the social work agency) and program evaluation
- Respondents' perceptions of the program implementation process
- Respondents' perceptions of program effectiveness, program success, and overall impression

In addition, the school was required to complete a school background information questionnaire with both closed- and open-ended questions to provide basic information about the school, Tier 1 Program implemented in the school, instructors conducting the Tier 1 Program, and the implementation process.

After data collection, the data were neatly typed, and the recordings of the interviews were transcribed and analyzed. The procedures for data collection described above satisfied Principle 3 in the implementation of evaluation research proposed by Shek et al.[18].

### Data Analyses

The qualitative data were analyzed using general qualitative analyses techniques[19]. There were three steps in the process. First, words, phrases, and/or sentences that formed meaningful units at the raw response level were coded. Second, with reference to the questions of the interview guide, the codes were further combined to reflect higher-order attributes at the category of codes level. Third, the categories of codes were further analyzed to reveal the broader themes at the thematic level. For example, the raw responses showing high support from the school contact person could be subsumed under the category of "support for the program and its rationales", which could be further subsumed under the broad theme of "school administrative arrangements and related issues" (see Table 1). Following the principles of qualitative analyses[18], the raw data and categorized data were kept in a systematic filing system in order to ensure that the findings were auditable.

## RESULTS

### Summary of the School Characteristics

As shown in Table 2, the interviewed school had participated in the Experimental Implementation Phase before and it joined the Tier 1 Program (Secondary 1 Curriculum) in the Full Implementation Phase. There were 119 Secondary 1 students in three classes. The 20-h full program (20 1-h sessions) was delivered in class teacher periods (11 h), Life Education classes (8 h), and project-based learning (1 h). In addition, the introduction session and conclusion session were arranged for students in the format of whole-form gatherings before and after the program implementation, respectively. The medium of instruction was Chinese. Because the school had a double class teachers system, both class teachers cooperated in running the program in the class teachers' period. In total, there were six class teachers, two subject teachers, one teaching assistant, and one social worker who conducted the Tier 1 Program in the school, but only three teachers and the social worker had participated in the training workshops arranged for the instructors before they implemented the program in the school.

**TABLE 1**
**Consolidated Interpretations on the Administrative Arrangements and Related Issues in the School**

| Aspect | Integrated Findings and Interpretations |
|---|---|
| (a) Support for the program and its rationales | • Strong support from the school contact person, which facilitated the program implementation.<br>• Support from experienced teachers exerted a positive influence on support and participation of other teachers.<br>• The teachers considered that the program was good for students, which facilitated the program implementation. |
| (b) Perceived program arrangement, and its advantages and disadvantages | • The program was arranged to fit into the existing school lessons with similar content, and the lessons were distributed according to the topics of the subjects.<br>• Advantages: (a) students valued the learning on positive development, (b) there was no need for much extra work to be undertaken.<br>• Disadvantage: Nil.<br>• Suggestions: some topics could be reallocated to other relevant subjects. |
| (c) Manpower deployment | • Systematic manpower deployment: one chief coordinator, a form coordinator, six class teachers, two specialist teachers, a social worker.<br>• Experienced and self-motivated teachers facilitated the implementation process. |
| (d) Facilities for implementation | • Sufficient equipment and facilities facilitated the implementation. |
| (e) Support within school and from social work agency | • Physical proximity facilitated the implementation.<br>• Attributes of the school principal: supportive and trusted her colleagues.<br>• Attributes of the school contact person: had leadership skills and clear direction, teacher-centered, supportive, motivational, and allowed collaborative decision making.<br>• Qualities of the teachers: cooperative, supportive, and had initiative to share experiences and information.<br>• Teachers perceived the support from social workers was sufficient and they were satisfied with it.<br>• The school had a good collaborative relationship with the social work agency. |
| (f) Impression on the overall administration | • The school contact person was satisfied with the administrative arrangement because he had justifications behind the adoption.<br>• Communication was good.<br>• Teachers had positive perceptions of the administration done by the school contact person. |
| (g) Advice for administrative arrangement for next academic year | • A systematic record system was desirable, although a simple recording system existed. |
| (h) Other aspects: meetings within school | • Set up a P.A.T.H.S. team to coordinate the related matters.<br>• A final evaluation meeting in term end.<br>• Periodic class management meetings among class teachers. |

The school arranged several meetings in the program implementation process. Before implementation, there were two preparation meetings, where the implementation objectives and arrangements were discussed. During implementation, the class teachers' meetings were held once every 2 months, in which teachers shared teaching experiences after classes. After implementation, there was an evaluation meeting, where issues related to preparation and implementation, the difficulties encountered, and follow-up were reported.

**TABLE 2**
**Summary of the Characteristics of the School**

| | |
|---|---|
| **I. Basic Information of the School** | |
| Total no. of Secondary 1 students (no. of Secondary 1 classes) | 119 (three classes) |
| Class teacher system | Double class teachers system |
| Participation in Life Education program organized by other organizations | No |
| No. of uniform/social and voluntary services groups in school | Six |
| Small groups, workshops, and seminars specifically arranged for S1 students | i. Environmental protection; ii. Dealing with Internet addiction; iii. S1 orientation |
| **II. Basic Information of Tier 1 Program Participation** | |
| Hours | 20-h full program |
| Mode | Mode 3 (20 1-h sessions) |
| Program arrangement | Class teachers' period (55%, about once every 2 weeks); |
| | Life Education (40%, about once every 2 weeks); |
| | Project-based learning (5%, once in first semester and once in second semester) |
| Rationales of the program arrangement | The topics related to individual aspects are implemented in school subjects, whereas the topics related to social aspects or group work are implemented in class teachers' period. |
| Medium of instruction | Chinese |
| Consolidation session | N.A. |
| Introduction session | Yes (whole-form gathering) |
| Conclusion session | Yes (whole-form gathering) |
| Supplementary activity | No |
| Other activities | No |
| Student assessment on P.A.T.H.S. Program | Yes (homework and examination in Life Education and project-based learning) |
| Have joined Experimental Implementation Phase | Yes |
| **III. Information of the Instructors Conducting Tier 1 Program** | |
| No. of instructors who conducted Tier 1 Program | Eight teachers, one teaching assistant, one agency social worker |
| No. of instructors who joined P.A.T.H.S. training workshops | Three teachers, two school social workers, and one agency social worker |
| No. of instructors who joined the workshops on "Handling Behavioral and Emotional Problems of Students" | Four teachers |
| No. of instructors who joined the workshops on "Personal Growth and Psychological Well-Being of Teaching Professionals" | None |
| **IV. Basic Information of Tier 1 Program Implementation** | |
| No. of preparation meetings and content | About two preparation meetings in class teachers' meetings, where there was discussion on the implementation aims, arrangement, and related matters |
| No. of instructors who conducted Tier 1 Program in each class | Two teachers (one agency social worker conducted some activities) |

*Table 2 continues*

**TABLE 2 (continued)**

| | |
|---|---|
| Person responsible for preparing the teaching materials and the way of preparation | The form coordinator read all curriculum instructions, made summaries for the teaching plans, and prepared all teaching materials, worksheets, and PowerPoint presentations for the class teachers. |
| Format and frequency of lesson preparation among instructors | The form coordinator prepared all teaching plans and the teachers discussed them together after receiving the teaching plans. |
| Format and frequency of experiences sharing after class among instructors | Once every 2 months in class teachers' meetings |
| No. of evaluation meetings and content | One final evaluation meeting, where there were reports on the issues related to preparation and implementation, the difficulties encountered, and follow-up |
| Cooperation between the school and social work agency | Social work agency provided social workers who offered advice and assisted some teaching |
| Role of the teacher in Tier 1 Program implementation | i. Teaching the program; ii. Monitoring classroom discipline; iii. Preparing teaching materials; iv. Coordinating the program-related matters; v. Follow-up |
| Role of the social worker in Tier 1 Program implementation | Coordinating the program-related matters |

In the implementation process, a form coordinator (the teaching assistant) was responsible for preparing summaries of all teaching plans and materials for the teachers. When teachers received the teaching plans, they discussed them together before running the activities in the classes. In the Tier 1 Program, the roles of teachers included teaching the program, monitoring classroom discipline, preparing teaching materials, coordinating the program-related matters, and following-up with the students. The social worker was mainly responsible for coordinating the program-related matters, but also would conduct some activities in classes.

## Administrative Arrangements of the School and Related Issues

As shown in Table 1, the findings on the school administrative arrangements and related issues were divided into several parts.

a. **Support for the program and its rationales** — The school contact person revealed that he considered that the Tier 1 Program was more interesting than other existing programs. He strongly supported the program and agreed to its rationales, and thus took initiative to arrange the program in his school. He also pointed out that the ready-made program materials and good reputation of the program also generated support among the teachers. Similar support from the teachers was observed, except that some teachers who had not participated in the training workshops expressed that they did not know the project rationales and the linkage among the Secondary 1, 2, and 3 curricula.
b. **Perceived program arrangement, including its advantages and disadvantages** — The school contact person perceived that the advantage of incorporating the program into the existing curriculum was to let the students learn about the subject matter and he could not perceive any disadvantage of such an arrangement. This view was shared by other teachers in the focus group, except that a teacher suggested the need to reallocate the topics on national identity to Integrated Humanities.
c. **Manpower deployment** — From the interviews, it was revealed that the school had arranged different parties who had specific strengths to implement the program. Along this manpower deployment, the school contact person perceived that different parties could complement each other and was happy that the experienced teachers worked on their own, which could lessen his coordination work.

d. **Facilities for implementation** — As revealed by the teachers, the facilities for program implementation in each classroom were sufficient. This is important because it made the teachers feel that it was easier to run the activities.
e. **Support within school and from social work agency** — As revealed from the interviews, there were several aspects of support within the school and from the social work agency in the implementation process. The above findings indicated that (i) physical proximity to each other in the school facilitated a supportive working environment for the teachers; (ii) the chief coordinator displayed the attributes of teacher-centeredness, motivation, collaborative decision making, leadership skills, and clear direction; (iii) psychological support from the principal was evident; (iv) having practical support from social workers and among the teachers was the key ingredient in program implementation.
f. **Impression on the overall administration** — The school contact person felt that he was quite satisfied with the administrative arrangement and communication among colleagues. The teachers also appreciated that the school contact person followed through the program implementation clearly and they were satisfied with the administration orchestrated by him. The above findings indicated that having justifications behind the timetable and manpower arrangement in the program implementation, communication, and support from school contact person were the main elements contributing to the satisfaction of the overall administration.
g. **Advice for administrative arrangement for the next academic year** — Although the school had simple records on the administrative procedure, the school contact person suggested that a systematic record system for administrative arrangement for next year be established so that a more apparent framework on the program arrangement and manpower deployment could be documented for future use whenever necessary.
h. **Other aspects** — Meetings within school: In the interview with the school contact person, he revealed that there were periodic class management meetings and a final evaluation meeting in school with a structured organization. The school set up a P.A.T.H.S. team, in which the school contact person was the chairman, and the members were the social workers and their supervisor, class teacher representatives, form coordinators, and Life Education teacher. At the end of the term, the team evaluated both the Tier 1 and Tier 2 Programs in each form as well as planned for next year's implementation. He also noted that the school carried out class management, i.e., the class teachers in each form had meetings every 2 months to discuss discipline, counseling, and class matters in that form. A representative was responsible for coordination and acted as a bridge with other school teams. As the P.A.T.H.S. Program was related to counseling, it was also discussed in the class teachers' meeting, and this was considered as periodic evaluation. The interpretations of these findings were that having a structured team, periodic meetings, and a final evaluation for program implementation were factors contributing to a smooth and organized implementation in the school. The integrated findings and interpretations are shown in Table 1.

## Implementation Characteristics in the School

The integrated findings and interpretations of the implementation characteristics can be seen in Table 3.

a. **Teachers' and students' responses in the implementation process** — The contact person pointed out that although the teachers were quite positive towards the program, they rarely openly expressed their appreciation and this characteristic might be related to the Chinese culture. About the students' responses in the implementation process, the teachers pointed out that the students considered the program to be quite good because they liked the audio-visual materials, activities,

**TABLE 3**
**Consolidated Interpretations on the Implementation Characteristics in the School**

| Aspect | Integrated Findings and Interpretations |
|---|---|
| (a) Teachers' and students' responses in the implementation process | • Teachers had positive comments on the program, but they rarely expressed their appreciation.<br>• Students liked activities and novel topics. Their interest and involvement varied, depending on the topics.<br>• Some students were not used to being open in sharing themselves. Some students were not cooperative in class. |
| (b) Coteaching | • Teachers cooperated and trusted each other in coteaching. |
| (c) Teaching techniques | • Technique 1: Encourage students to speak.<br>• Technique 2: Teach and motivate students according to their qualities and standards.<br>• Technique 3: Recognize positive behaviors.<br>• Technique 4: Check students' assignments and give feedback.<br>• Technique 5: Teachers' personal sharing. |
| (d) Handling students' worksheets | • Checking students' assignments was beneficial.<br>• Completed worksheets needed to hand in.<br>• Folders were used. |
| (e) Student assessment in the Tier 1 Program | • Tier 1 Program assessment focused on application was carried out for Life Education and project-based learning.<br>• No examination for class teachers' period. |
| (f) Difficulties encountered and solutions | • The school contact person did not perceive any big difficulties, but felt exhausted due to heavier workload. He revealed minor difficulties that had been solved.<br>• Teachers' difficulties: perceived insufficient time to run activities, perceived strange linkage between some topics, program inflexibility.<br>• Solutions: to familiarize oneself with the program, have prior experience, and good preparation. |
| (g) Advice for next year's implementation | • Prepare early, periodically update the teaching materials, and search for more relevant and the latest information to meet students' needs. |

debates, and role play. However, the students were not receptive to sharing with teachers and some of them were not involved in the lessons. The teachers also pointed out that students were interested in novel topics and they felt bored when attending intensive sessions.

b. **Coteaching** — The focus group interview findings showed that the teachers were supportive to one another and cooperated well in coteaching.

c. **Teaching techniques** — Several techniques were used by the teachers in the program implementation process. The teachers felt that it was important to allow students to have freedom of speech, to know the qualities and preferences of the class students (to motivate their participation accordingly), to recognize positive behaviors, to be open to sharing personal experiences, and to be serious in checking the students' work and performance, and give feedback.

d. **Handling students' worksheets** — The teachers felt that the completed worksheets should be handed in and kept in the students' folders neatly. The collections could help the students to reflect on their learning and growth, and teachers need to act as monitors and facilitators in the process. In addition, the teachers' seriousness in checking the students' work and giving feedback were crucial because they motivated students to complete their work seriously.

e. **Student assessment in the Tier 1 Program** — The school contact person reported that there were examinations in Life Education and project-based learning. However, the examination content was similar to, but not exactly the same as, the content of the P.A.T.H.S. Program and it tested application, but not memorization. On the other hand, the teachers reported that there was no examination for the topics covered in the class teachers' period and a teacher considered that the students' reflection in the growth puzzles was a kind of student self-assessment.

f. **Difficulties encountered and solutions** — From the findings, it was noted that the school contact person did not perceive any big difficulties; he had been to the training workshops and he was familiar with the program. However, the front-line teachers encountered more difficulties in running the activities in classroom, and nonparticipation in the training workshops might be one of the reasons for their frustration. Hence, familiarization with the program, prior experience, and good preparation were solutions to the difficulties encountered in the program implementation. In addition, attending training workshops is important because teachers can familiarize themselves with the program rationales, content, and teaching strategies.

g. **Advice for next year's implementation** — In the focus group interviews, the teachers suggested that early preparation, possession of adequate information, and periodic updating of the teaching materials to accommodate the students' needs was relevant advice for next year's implementation. In addition, it was felt that teachers needed to be responsible and student-centered in the program implementation.

## Perceived Program Effectiveness, Program Success, and Overall Impression

The integrated findings and interpretations can be seen in Table 4.

a. **Perceived program effectiveness** — The school contact person said that he knew the least on the program effectiveness because he had not taught Secondary 1 students. Similarly, the teachers were unsure about changes in students specifically brought forth by the program because there were many other factors influencing the students' growth. They suggested that objective assessment and long-term student follow-up were important reference points for determining program effectiveness.

b. **Perceived program success** — The contact person perceived that the program was successful if "success" referred to the students' improvement from the original baseline. A teacher also perceived the program to be successful as reflected from the students' involvement and program design. On the other hand, some teachers did not directly respond to the question of program success, but they considered the program to be beneficial to students. They also perceived the program to be a good opportunity for students to reflect on the topics and for teachers to get acquainted with the students. These findings showed that different people had different definitions of success, which generated different answers to the questions of perceived program success.

c. **Overall impression** — The contact person had a positive impression and no negative comments on the program. He perceived the program as diverse, flexible, and helpful to students, and thus teachers were pleased to implement. He felt exhausted and he had difficulty in arranging teachers to attend the training workshops. Similarly, many teachers had positive impressions of the program because they perceived that adolescents needed such a program, and the program was good, had diverse activities, and could give positive messages to adolescents. The interpretations of the above findings were that teachers enjoyed teaching only when the students responded, and such teacher-student interaction was a kind of encouragement and teaching motivation. On the other hand, teachers had negative feelings when they felt stressed and exhausted. Particularly, teachers did not enjoy the program if they perceived teaching to be a chore and not a means to facilitate the students' development.

**TABLE 4**
**Consolidated Interpretations on the Perceived Program Effectiveness, Program Success, and Overall Impression**

| Aspect | Integrated Findings and Interpretations |
|---|---|
| (a) Perceived program effectiveness | • The contact person did not think he was in an appropriate position to judge the program effectiveness.<br>• Some teachers were unsure about the changes.<br>• Objective assessment and long-term follow-up are important in order to assess program effectiveness. |
| (b) Perceived program success | • The contact person had no clear answer on effectiveness, but perceived the program to be successful if students had improvement.<br>• Another teacher perceived it was successful, which was reflected from student involvement and program design.<br>• Some teachers perceived the program as beneficial to students; it was a good opportunity for students to come across the subject matter, for students' learning, and for teachers to get acquainted with students.<br>• Different people had different definitions of success. |
| (c) Overall impression | • Most of the respondents had positive impressions of the program.<br>• Teachers enjoyed teaching only when the students responded. The interaction was a kind of encouragement and teaching motivation.<br>• Teachers had negative feelings when they felt stressful and exhausted.<br>• Teachers did not enjoy the program if they perceived teaching to be a chore, not a means to facilitate students' development. |

## DISCUSSION

Because there are very few case studies that examine program implementation of positive youth development programs, the present case study is a good attempt to illustrate how the Tier 1 Program of the Project P.A.T.H.S. could successfully be implemented in the school context. It also generates implications regarding future program implementation.

### Factors that Facilitate or Impede Program Implementation

With reference to the 5 "P"s (i.e., program, people, process, policy, and place) in the conceptual framework, factors that facilitate or impede program implementation are presented in Table 5.

First, echoing previous findings, the present study showed that when the instructors perceived the program to be comprehensive and the activities met the developmental needs of the students, they would be more willing to implement the program in the school. Most importantly, the perception of having ready-made and helpful teaching manuals was the facilitating factor that induced the instructors to deliver the program willingly, whereas perceived inflexibility in using the ready-made materials, having too many activities to be implemented in limited class time, and inadequate variety in the activity format were the obstacles in program implementation.

Second, as the teachers are the front-line instructors of the program, they have a very crucial role in the program implementation. This study showed that if the instructors were trained and familiar with the program rationales, they would be more ready to implement the program, which in turn raised the implementation quality. In addition, this study showed that the teachers' qualities, such as being student-

**TABLE 5**
**Factors that Facilitate the Implementation Process and Obstacles Affecting the Quality of Implementation**

| Aspect | Factors that Facilitate the Implementation Process | Obstacles Affecting the Quality of Implementation |
|---|---|---|
| (a) Program | • Comprehensive program<br>• Activities matching students' developmental needs<br>• Perceived helpfulness<br>• Diverse activities<br>• Ready-made teaching manuals | • Perceived having too many activities within a 1-h lesson<br>• Perceived doubtful linkage between some topics<br>• Perceived inadequate variety in the activity format<br>• Perceived insufficient flexibility (e.g., cannot add additional information to accommodate unique students' needs) |
| (b) People | • Support for the program and appreciation for the program rationales<br>• Student-centered, responsible<br>• Passionate and motivated to implement the program<br>• Prior experience in running similar programs<br>• Trust and cooperation among instructors<br>• School principal is supportive<br>• The chief coordinator has leadership skills and clear direction, is teacher-centered and motivational<br>• Students are motivated to learn | • Unfamiliar with the program and its rationales<br>• Received no training<br>• Task-oriented<br>• Lack of appreciation<br>• Some students are less motivated and involved |
| (c) Process | • Good collaborative relationship between school and social work agency<br>• Adequate support from social work agency<br>• Coteaching<br>• Good preparation before teaching<br>• Varied teaching techniques (e.g., to encourage students to speak, to teach and motivate students according to their quality, to recognize positive behavior, to share personal experiences, to be serious)<br>• Worksheets are handed in and kept in students' folders<br>• Check students' assignments and give feedback<br>• Good communication | • Insufficient time to run the program<br>• Do not have periodic updating of the teaching materials<br>• No long-term follow-up for students |
| (d) Policy | • Justifications for the program arrangement and manpower deployment<br>• Specialist teaching<br>• Structured teams and person-in-charge, with specific roles and responsibilities<br>• Meetings and evaluation on program implementation<br>• Documentation of administrative procedure and record requirement<br>• Collaborative decision making<br>• Buy-in from the teachers | • No objective assessment and evaluation on program effectiveness |

*Table 5 continues*

**TABLE 5 (continued)**

| | | |
|---|---|---|
| (e) Place | • Sufficient hardware equipment for running activities<br>• Physical proximity that enables teachers' communication<br>• Dedicated school culture | Nil |

centered, responsible, passionate, and motivated to implement the program, were key factors in engaging the students and enabling the implementation quality. Moreover, having prior experience in running similar programs, and having supportive and cooperative colleagues, were assets that enhanced the program delivery. Furthermore, this study showed that the seeming lack of appreciation from instructors, which may be due to the Chinese culture, may affect teaching motivation and quality.

Besides the qualities of instructors, it is widely recognized that support from the school principal and clear direction from the coordinator were the main facilitating factors of program implementation quality. This study further showed that the principal and the coordinator should be teacher-centered and motivated because these conditions would enhance teacher devotion to the program implementation process. Finally, results showed that students were other key persons in the program implementation because they were expected to be active learners in experiential learning. If students were less motivated and less involved, it would affect the instructors' motivation and the learning atmosphere, thus influencing the implementation quality.

Third, the present findings suggest that it is important to have varied teaching techniques when delivering the program. There are various teaching techniques that can be used in the class teachers' period, such as encouraging students to speak their mind and having more interaction with the students. The present study also suggests that students are more motivated to learn when the instructors are open to sharing their personal experiences and able to recognize the students' positive behavior. In addition, instructors need to check the students' assignments and give feedback so as to encourage the students to be serious learners. The students' worksheets are better handed in and kept neatly in student folders so that the students can review their learning and development after the completion of the program. All these teaching techniques not only facilitate teaching, but also strengthen the rapport between class teacher and the students.

In addition, although the lesson can be run by one instructor per class, coteaching is good if the teachers can collaborate with each other because it can lower the teacher-student ratio and ensure that the students' needs are better met. Furthermore, good preparation and sharing among colleagues before lessons can improve the program implementation quality. In addition, if the instructors often cut the activities when they perceive that they are running out of time, it will seriously affect the students' learning.

Fourth, to implement the program in the class teachers' period, the school needs to have a clear policy regarding the practical arrangement and respective manpower deployment. The school can also set up a team for running the program in which the people involved have specific roles and responsibilities and they can make collaborative decisions. This study further showed that documentation of the administrative procedure and record are needed in order to enhance smooth implementation of the program. Having regular meetings and final evaluation on the program implementation is also necessary for further improvement. In contrast, having no objective assessment and evaluation on the program effectiveness would be obstacles in the assessment of the program failure or success.

Finally, it is necessary to have a dedicated school culture in order to run a positive youth development program. The school can promote and encourage such a culture by creating physical proximity for the class teachers to sit and work together so that they can communicate, share experiences, and support each other in the program implementation. In addition, the school should have sufficient hardware equipment in each classroom for instructors to run the activities, which can then lessen the instructors' workload in arranging teaching equipment and will allow them to have more time for teaching preparation.

## Implications of the Findings for Future Implementation of the Program

Based on the present findings and analyses, several implications for future program implementation were generated. A summary is shown in Table 6. There are several implications for school administrative arrangements. First, it is suggested that the program be incorporated into the existing school subjects, with similar content and topics, because this can help to avoid adding extra workload for the instructors and help students value the learning. Second, it is recommended that the school formulates an organized manpower deployment plan and sets up a structured team for program implementation with designated roles and responsibilities. Together with this, good communication among different parties is needed. Third, it is necessary to create supportive and cooperative working relationships within the school, and to have good collaboration between the school and social work agency. Fourth, it is suggested that periodic meetings and evaluation on program implementation be held, and that the success and failure of program implementation be documented via a systematic record system because this can facilitate the ongoing program implementation, as well as that of the implementation in the following years. Fifth, the school needs to have sufficient equipment and facilities for instructors to run the activities easily. Sixth, the instructors should attend training workshops to help familiarize themselves with the program and teaching techniques, and to change their myths of program inflexibility. This could also enable the instructors to internalize the program philosophy and to strengthen their motives to teach this kind of program. Lastly, the school needs to organize objective assessments for evaluating the program effectiveness.

In the aspect of implementation process, there are several implications. First, various teaching techniques are needed to stimulate teaching and learning, such as encouraging students to speak, teaching and motivating students according to their qualities, recognizing positive behavior, sharing personal experiences, and checking the students' assignments. In handling the students' worksheets, feedback should be given and folders can be used to keep the students' worksheets neatly. Also, instructors can encourage students to use the growth puzzles to reflect and assess their growth. Second, coteaching is desirable for running activities in the classroom, although the designed activities could be run by one instructor only. Third, instructors should be responsible and student-centered. Having prior experience, good preparation, periodic updating of the teaching materials, and possession of good teaching skills can enhance teaching qualities. Fourth, noting that interaction can act as a motivator for teachers, instructors need to encourage students to be open while participating in sharing and eagerly involved in the activities. Fifth, as students with psychosocial needs require more attention and nurturing, instructors need to follow up with needy students. Finally, instructors should be given the opportunity to show appreciation for the program because Chinese people may hesitate to do so although they have positive comments on the issues.

## Limitations of the Case Study

There are some limitations of the present study. First, as only one nonrandomly selected school was involved in this case study, generalization of the findings is questionable. Nonetheless, documentation of the school's successful experiences of the program implementation can still be taken as a reference. Second, as the present interviews only involved a school contact person and six class teachers, the data collected would be more thorough if interviews with the school principal, form coordinator, and social worker were involved. Third, the case study was conducted in a retrospective manner, and it would be more valuable if periodic interviews with the instructors and observations of the program delivery in classes were conducted as well. Fourth, in the data analyses, coding and categorization were done by the second author and cross-checked by the first author. Although this method was justified for a small-scale case study, inter- and intrareliability was not conducted. Fifth, the presence of interviewers may have affected the responses of the respondents (e.g., social desirability effect) in face-to-face interviews. However, as each interview was conducted for about 90 min and follow-up calls were arranged, this method of data collection was justified.

**TABLE 6**
**Implications of the Findings for Future Implementation**

| Aspect | Implications |
|---|---|
| **(a) School administrative arrangement** | • To incorporate the program into existing school subjects and match the topics of the subjects.<br>• To have an organized manpower deployment plan, and to set up a structural team for program implementation with specific roles and responsibilities.<br>• To have good communication.<br>• To create a supportive and cooperative working relationship within the school, and between the school and social work agency.<br>• To have periodic meetings and evaluation on the program implementation.<br>• To document the success and failure of program implementation, via a systematic record system.<br>• To have sufficient equipment and facilities in classrooms and schools.<br>• Instructors should attend training workshops to familiarize themselves with the program and teaching techniques, and to change their myths of program inflexibility.<br>• Instructors need buy-in to the program rationales and have motivation to teach this kind of program.<br>• Objective assessment is needed to assess the program effectiveness. |
| **(b) Implementation process** | • Coteaching is desirable for running activities in the classroom, although the designed activities could be run by one instructor only.<br>• Various teaching techniques are needed to stimulate teaching and learning, including (a) to encourage students to speak out, (b) to teach and motivate students according to their qualities, (c) to recognize positive behavior, (d) to share personal experiences, and (e) to be serious.<br>• Instructors should check students' assignments, give feedback.<br>• Folders are used to keep students' worksheets neatly.<br>• Instructors should encourage students to use the growth puzzles to reflect and assess their growth. More varied ways to engage students.<br>• Instructors need to have prior experience, good preparation, periodic updating of the teaching materials, and be informative.<br>• Instructors need to be responsible and student-centered.<br>• Instructors need to conduct follow-up with students.<br>• Instructors should be given the opportunities to show appreciation of the program.<br>• Students are encouraged to be open while participating in sharing and giving responses to instructors, as interactions can act as a teaching motivation. |

Finally, regarding perceptions of the program effects, while the conclusions on program effectiveness were not definitive, perceptions of program success and overall impressions were generally positive. In future, more attempts should be made to clarify this issue.

Despite the above limitations, the present findings are generally coherent with the evaluation findings generated from different sources and different methods, including objective outcome evaluation[20], subjective outcome evaluation[21,22,23,24], qualitative evaluation[25,26], process evaluation[27,28], and interim evaluation[3,29], which suggest that the Tier 1 Program (Secondary 1 Curriculum) of the Project P.A.T.H.S. is effective and well received by the different stakeholders of the program.

## ACKNOWLEDGMENTS

The preparation for this paper and Project P.A.T.H.S. were financially supported by The Hong Kong Jockey Club Charities Trust.

## References

1. Shek, D.T.L. (2006) Construction of a positive youth development program in Hong Kong. *Int. J. Adolesc. Med. Health* **18,** 299–302.
2. Shek, D.T.L. and Ma, H.K. (2006) Design of a positive youth development program in Hong Kong. *Int. J. Adolesc. Med. Health* **18(3),** 315–327.
3. Shek, D.T.L., Ma, H.K., and Sun, R.C.F. (2008) Interim evaluation of the Tier 1 Program (Secondary 1 Curriculum) of the Project P.A.T.H.S.: first year of the full implementation phase. *TheScientificWorldJOURNAL* **8,** 47–60.
4. Fagan, A.A. and Mihalic, S. (2003) Strategies for enhancing the adoption of school-based prevention programs: lessons learned from the blueprints for violence prevention replications of the life skills training program. *J. Community Psychol*. **31(3),** 235–253.
5. Elias, M.J., Gager, P., and Leon, S. (1997) Spreading a warm blanket of prevention over all children: guidelines for selecting substance abuse and related prevention curricula for use in the schools. *J. Prim. Prev*. **18(1),** 41–69.
6. Kumpfer, K.L. and Alvarado, R. (2003) Family-strengthening approaches for the prevention of youth problem behaviors. *Am. Psychol*. **58(6/7),** 457–465.
7. Liljia, J., Wilhelmsen, B.U., Larsson, S., and Hamilton, D. (2003) Evaluation of drug use prevention programs directed at adolescents. *Subst. Use Misuse* **38(11–13),** 1831–1863.
8. Nation, M., Crusto, C., Wandersman, A., Kumpfer, K.L., Seybolt, D., Morrissey-Kane, E., and Davino, K. (2003) What works in prevention: principles of effective prevention programs. *Am. Psychol*. **58,** 449–456.
9. Kam, C.M., Greenberg, M.T., and Walls, C.T. (2003) Examining the role of implementation quality in school-based prevention using the PATHS curriculum. *Prev. Sci.* **4(1),** 55–63.
10. Ennett, S.T., Ringwalt, C.L., Thorne, J., Rohrbach, L.A., Vincus, A., Simons-Rudolph, A., and Jones, S. (2003) A comparison of current practice in school-based substance use prevention programs with meta-analysis findings. *Prev. Sci*. **4(1),** 1–14.
11. Callahan, B.M., Benton, S.L., and Bradley, F.O. (1995) Implementing a drug prevention program: a comparative case study of two rural Kansas schools. *J. Alcohol Drug Educ*. **41,** 26–48.
12. Ringwalt, C.L., Ennett, S., Johnson, R., Rohrbach, L.A., Simons-Rudolph, A., Vincus, A., and Thorne, J. (2003) Factors associated with fidelity to substance use prevention curriculum guides in the Nation's middle schools. *Health Educ. Behav*. **30,** 375–391.
13. Rohrbach, L.A., Graham, J.W., and Hansen, W.B. (1993) Diffusion of a school-based substance abuse prevention program: predictors of program implementation. *Prev. Med*. **22,** 237–260.
14. Rohrbach, L.A., D'Onofrio, C.N., Backer, T.E., and Montgomery, S.B. (1996) Diffusion of school-based substance abuse prevention programs. *Am. Behav. Sci*. **39(7),** 919–934.
15. Eccles, J. and Gootman, J.A. (2002) *Community Programs to Promote Youth Development*. National Academy Press, Washington, D.C.
16. Yin, R.K. (2003) *Applications of Case Study Research*. Sage, Thousand Oaks, CA.
17. Yin, R.K. (2003) *Case Study Research: Design and Methods*. Sage, Thousand Oaks, CA.
18. Shek, D.T.L., Tang, V., and Han, X.Y. (2005) Quality of qualitative evaluation studies in the social work literature: evidence that constitutes a wakeup call. *Res. Soc. Work Pract*. **15,** 180–194.
19. Miles, M.B. and Huberman, A.M. (1994) *Qualitative Data Analysis*. Sage, Thousand Oaks, CA.
20. Shek, D.T.L. (2006) Effectiveness of the Tier 1 Program of the Project P.A.T.H.S.: preliminary objective and subjective outcome evaluation findings. *TheScientificWorldJOURNAL* **6,** 1466–1474.
21. Shek, D.T.L. and Ma, H.K. (2007) Subjective outcome evaluation of the Project P.A.T.H.S.: findings based on the perspective of the program participants. *TheScientificWorldJOURNAL* **7,** 47–55.
22. Shek, D.T.L., Siu, A., and Lee, T.Y. (2007) Subjective outcome evaluation of the Project P.A.T.H.S.: findings based on the perspective of the program implementers. *TheScientificWorldJOURNAL* **7,** 195–203.
23. Shek, D.T.L. and Sun, R.C.F. (2007) Subjective outcome evaluation of the Project P.A.T.H.S.: qualitative findings based on the experiences of program participants. *TheScientificWorldJOURNAL* **7,** 686–697.
24. Shek, D.T.L. and Sun, R.C.F. (2007) Subjective outcome evaluation of the Project P.A.T.H.S.: qualitative findings based on the experiences of program implementers. *TheScientificWorldJOURNAL* **7,** 1024–1035.
25. Shek, D.T.L., Lee, T.Y., Siu, A., and Lam, C.M. (2006) Qualitative evaluation of the Project P.A.T.H.S. based on the perceptions of the program participants. *TheScientificWorldJOURNAL* **6,** 2254–2263.
26. Shek, D.T.L., Sun, R.C.F., Lam, C.M., Lung, D.W.M., and Lo, S.C. (2008) Evaluation of Project P.A.T.H.S. in Hong Kong: utilization of student weekly diary. *TheScientificWorldJOURNAL* **8,** 13–21.

27. Shek, D.T.L., Ma, H.K., Lui, J.H.Y., and Lung, D.W.M. (2006) Process evaluation of the Tier 1 Program of the Project P.A.T.H.S. *TheScientificWorldJOURNAL* **6,** 300–309.
28. Shek, D.T.L., Ma, H.K., Sun, R.C.F., and Lung, D.W.M. (2008) Process evaluation of the Tier 1 Program (Secondary 1 Curriculum) of the Project P.A.T.H.S.: findings based on the full implementation phase. *TheScientificWorldJOURNAL* **8,** 35–46.
29. Shek, D.T.L. and Sun, R.C.F. (2006) Implementation of the Tier 1 Program of the Project P.A.T.H.S.: interim evaluation findings. *TheScientificWorldJOURNAL* **6,** 2274–2284.
